# The Influence of the Presentation of Camera Surveillance on Cheating and Pro-Social Behavior

**DOI:** 10.3389/fpsyg.2018.01937

**Published:** 2018-10-16

**Authors:** Anja M. Jansen, Ellen Giebels, Thomas J. L. van Rompay, Marianne Junger

**Affiliations:** ^1^Department Psychology of Conflict, Risk and Safety, Faculty of Behavioral Sciences, University of Twente, Enschede, Netherlands; ^2^Department of Communication Science, Faculty of Behavioral Sciences, University of Twente, Enschede, Netherlands; ^3^Department of Industrial Engineering and Business Information Systems, School of Management and Governance, University of Twente, Enschede, Netherlands

**Keywords:** CCTV, mirrors, self-monitoring, observation, cheating behavior, crime prevention

## Abstract

**Introduction:** This study is aimed at gaining more insight into the effects of camera-surveillance on behavior. It investigates the effects of three different ways of “framing” camera presence on cheating behavior and pro-social behavior. First, we explore the effect of presenting the camera as the medium through which an intimidating authority watches the participant. Second, we test the effect of presenting the camera as being a neutral, non-intimidating viewer. Third, we investigate the effect of watching oneself via a camera. In contrast to most studies on camera surveillance, we will conduct our experiments in an indoor setting. We also explore possible interaction effects of personality traits; we measured Locus of Control, Need for Approval, Self-Monitoring and Social Value Orientation.

**Methods:** In this experiment participated 86 students, randomly distributed over four conditions: three different ways of framing the camera presence, plus a control condition. Our main dependent variables were various kinds of cheating and pro-social behavior. We established the participant's relevant personality traits using a classification tree.

**Results:** For cheating behavior, findings showed that in the “authorative” way of framing camera presence and in the situation in which participants viewed themselves, participants cheated significantly less compared to a situation without camera-surveillance. We did not find significant effects of camera surveillance on pro-social behavior. Looking at personality traits, we found an indication that people with an internal locus of control are more inclined to cheat when there is no camera present compared to people with an external locus of control. However, the effects of our manipulations were stronger.

**Conclusion:** Our findings support the idea that the framing of a camera's presence does indeed influence cheating behavior, adding to the preventive effects of camera-surveillance. Additionally, this study provides some valuable insights into the influence of camera presence on behavior in general.

## Introduction

Camera surveillance is widely used in western countries, and its use is ever increasing. Cameras are often placed as a means to ensure that criminals and vandals are easier to trace, but according to Welsh and Farrington (2009), camera surveillance could also be seen as an instrument of situational crime prevention. The question is, however, to what extent the presence of cameras truly has a preventive effect on undesirable behavior, and whether a possible preventive effect would also apply to camera surveillance in different environmental situations, specifically in indoor settings.

Various studies have also shown that the preventive effect of cameras on undesirable and criminal behavior is not straightforward. For example, the meta-analyses of Welsh and Farrington (2002, 2009) show divergent results (i.e., in some places or situations cameras do seem to work, in other situations they do not), calling into question whether camera surveillance should be considered a dependable method of crime prevention. These meta-analyses show that the (environmental) context is an important factor to reckon with; for example, camera surveillance seems more effective in car parks compared to other settings (such as town centers and public housing communities). Studying how and why camera surveillance works and whether the presentation of, and notifications about, the camera surveillance influences its effectiveness might give more insight in the reasons why these meta-analyses show such a wide range of results.

Of particular relevance in this context are long-standing research findings from the social and behavioral sciences which testify to the importance of “framing”; the presentation and communications surrounding objects and information, possibly changing the attitudes of the public toward it (see Nelson et al., 1997). In the current research we will focus on the communications surrounding camera surveillance, aimed at informing the public about the camera's purpose. Framing of camera surveillance is often implemented by signs or posters informing the public about the presence of surveillance cameras, or by voice messages; for instance at train or metro stations. These signs, posters or messages may differ on various aspects (e.g., length or specificity), but arguably one of the most important factors influencing the effectiveness of camera surveillance involves whether or not the camera surveillance is presented in an intimidating or friendly manner to the public, or if awareness of their behavior is invoked. For instance, signage may stress the notion of being watched by an authority in an intimidating manner, or it may rather convey friendliness or helpfulness and stress the notion that CCTV is there for civilian safety.

Accordingly, one way of furthering insights into the effectiveness of camera surveillance might involve differentiation between different ways in which CCTV is framed to the public. Surprisingly, the influence of the framing of camera surveillance on the effectiveness of camera surveillance in influencing undesirable behavior has not been researched as of yet, yet it could be a valid explanation for the pronounced differences found in the effectiveness of CCTV in different studies. In the current study we examine three types of framing originating from different theoretical perspectives in a controlled experimental setting. As such, we explore and integrate theories from criminology with theories from social and environmental psychology. Additionally, we will focus on both interpersonal and intrapersonal processes to gain further insights into effects of camera presence and framing thereof on behavior.

In this study we will focus our examination on indoor settings. As mentioned before, many studies on the effectiveness of camera surveillance are conducted in outside, public spaces (see Welsh and Farrington, 2009). However, in recent years, cameras are also increasingly used in indoor settings such as shops, casinos, public spaces, governmental institutions, office environments and educational settings. In these type of settings, people often perform tasks or activities where cheating might occur (e.g., when completing a test or filling out a tax form). Importantly, the presence of camera surveillance in indoor environments might influence such unwarranted types of behavior. Additionally, people often interact and assist each other in these types of settings, and the presence of the watching eye of a camera might have an effect on pro-social behavior too. However, as of yet, research assessing effects of CCTV in indoor areas on these types of behavior is scarce.

A notable use of indoor camera surveillance is within the home of students enrolled in online courses, in order to prevent cheating during electronic exams (Eisenberg, 2013). In fact, some online learning institutions, like Coursera, offer courses (taught by professors from various prestigious universities) through which students can earn actual college credits. This raises the question how accurate the results of electronic tests are when they are taken from the student's home. There are several methods of preventing cheating at electronic exams, including camera feeds watched remotely, capturing and streaming the screen of the students, and recording keystrokes and mouse clicks (Sarrayrih and Ilyas, 2013; Bawarith et al., 2017).

When contemplating differences between indoor settings (including office buildings or in family home settings) and outdoor surveillance (e.g., streets or secluded parking garages), the feeling of being watched is arguably one of the most distinguishing features. That is, people would feel more like they are being watched or observed in indoor environments compared to outdoor environments, which could reduce the preventive effect of camera surveillance (often attributed to this feeling of being watched). Arguably (and of particular relevance to the current research) such perceptions and feelings are dependent on how camera presence is framed by means of notifications or information messages accompanying CCTV. Manipulating the context of the camera surveillance could increase its effectiveness. Additionally, implementing and manipulating the notifications accompanying the presence of the cameras is more straightforward and more noticeable in an indoor setting than in an outdoor setting.

In addition to preventing crimes such as shoplifting or vandalism, in indoor settings, smaller (but nonetheless troubling) crimes such as cheating and fraud (e.g., filling out incorrect or false information on forms or tests) and antisocial behaviors (e.g., being rude and unwilling to help others) may also be targeted. In the current undertaking we will therefore study effects of camera surveillance on cheating and occurrences of pro-social behaviors.

### The current research

Central to this research is the notion that cheating can be seen as a type of dishonest behavior to further selfish goals. Cheating has many parallels to other types of dishonest behavior: cheating is immoral, is being frowned upon by others, and an offender will be sanctioned when caught (Tittle and Rowe, 1973). In our study, participants could earn extra money dishonestly if they decided to cheat, so in this case cheating could be seen as a variant of stealing. As for pro-social behavior, three different types of behavior were studied. We used the cleaning up of (their own) trash, which has been studied extensively before, to be able to compare our findings with other studies. Additionally, we used a voluntary donation for a good cause, because charitable giving has also been used previously as a measure of pro-social behavior (e.g., Twenge et al., 2007; Van Rompay et al., 2009). Finally, we studied helping behavior by investigating the extent to which participants helped out the experimenter with picking up dropped items from the floor after a minor mishap (a similar measure as used in Twenge et al., 2007, and in Van Rompay et al., 2009, where participants could help an experimenter to collect dropped papers, either with or without a security camera present). Before presenting further details of this study, we will first elaborate on the processes underlying camera surveillance, drawing both from criminal and psychological frameworks.

### A multi-disciplinary approach to camera surveillance

While criminological theories focus mainly on the prevention of criminal behaviors such as vandalism and theft, most psychological theories assume a broader spectrum of behavior that might be influenced by the presence of cameras. For example (in a social psychological approach), the social impact theory by Latané (1981) states that the real or imagined presence of others can be regarded as a social force, affecting emotions, impressions, values and ultimately, behavior. Expanding on this reasoning, camera presence could affect both undesired behavior as well as desired, pro-social behavior.

In the current study we will examine various types of cheating in addition to three different types of pro-social helping behavior; namely cleaning one's trash, helping another person and donating to a charity.

The first approach we take in investigating how camera surveillance can affect behavior focuses mainly on the prevention of undesired behavior, and is based on the Rational Choice perspective of (Clarke and Felson, 1993) (also see Cornish and Clarke, 1986, 2008). This criminological perspective assumes that most criminal behavior is the result of a rational assessment of the costs and benefits of a certain action. Based on that perspective, people would only engage in deviant behavior when the benefits outweigh the costs (Cornish and Clarke, 1986, 2008; Braga, 2010). Since the presence of security cameras enhances the risk of being caught, this should lead to a decline in deviant behavior. This consideration is also evident in the Routine Activity Theory (Cohen and Felson, 1979; Felson, 1998, 2006), which is a derivative of the Rational Choice perspective. The Routine Activity Theory states that one of the three factors that could prevent the occurrence of a criminal incident is the presence of a capable guardian. According to the Social Control Theory (Hirschi, 2002), which is a complementary approach, this capable guardian should be perceived as an authority figure who has the ability to punish, thus placing strong emphasis on the probability of detection and the risk of punishment. Many studies do indeed suggest that deterrence caused by the threat of punishment is an important factor in reducing crime (e.g., Akers, 1990; Braga and Weisburd, 2012). The effect of an authority watching is demonstrated in a study by Sigelman and Sigelman (1976), which shows that the presence of an uniformed authority figure significantly decreases rates of traffic violations.

The presence of a security camera could fulfill the role of a guardian, since camera surveillance is a channel through which authority figures could monitor actions or events unfolding, leading to increased risk of detection and punishment. Levine (2000) did indeed state in his study on the effects of security cameras on public behavior that -in order for camera surveillance to be effective-, people should (1) be aware they are being monitored, (2) know who is watching, and (3) know which types of behaviors are punishable. Following this line of reasoning, it is thus important that camera surveillance is salient, and framed in such a way so that people have the impression that an authority figure is watching, who has the ability to punish deviant behavior.

Interestingly, Van Bommel et al. (2014) have found a surprising interaction effect of camera presence on helping behavior: without bystanders present, people seemed to intervene less often when money was stolen from a (confederate) victim when a security camera was present. This implies that individuals become *less* helpful in the presence of authoritative camera surveillance. Levine (2000) suggests this is the case because people feel less responsible to help others when they think an authority is keeping watch, a principle referred to as “diffusion of responsibility” (Darley and Latan, 1968). Together, this leads to the following hypotheses:

*Compared to a control situation without camera presence, both cheating (1a) and pro-social behavior (1b) will be discouraged when camera surveillance is presented in a salient and intimidating manner, enhancing the notice of a watchful authority capable of punishment*.

The second approach we take in order to explore how camera surveillance might influence behavior is based on psychological theories, mostly focusing on pro-social behavior and interpersonal processes. We mentioned before that the social impact theory by Latané (1981) implies that even the imagined presence of others (i.e., when a camera is “watching”) can be regarded as a social force, ultimately affecting behavior. A possible explanation for this phenomenon is that the presence of others (real, anticipated or imagined) may induce the feeling of being evaluated, encouraging self-evaluation and impression-management (Leary and Kowalski, 1990), which might result in behavior adjustments with the aim of presenting a socially-desirable image to the outside world. A typical category of norm-congruent behavior that is likely to carry away others' approval, is pro-social behavior (Kallgren et al., 2000), or “helping others.” If the presence of a camera evokes the feeling of being evaluated by others, pro-social behavior might be encouraged.

Govern and Marsch (2001) do indeed state that camera presence might encourage people to represent themselves well to an audience, presumably because a watchful eye increases self-awareness in their study. Similarily, Van Rompay et al. (2009) suggest that people observed by cameras feel that their behavior is being evaluated, and hence might adjust their behaviors in accordance with social norms. Findings from their study indeed showed that people were more willing to assist others when a camera was present and visible. Further underscoring the importance of impression management behaviors in relation to camera surveillance, findings further showed that this effect only surfaced for people with a strong need for the approval of others, and only in relation to behaviors that can actually be observed by cameras.

Based on the Social Impact Theory (Latané, 1981) we assume that undesired behavior would likewise be affected when a camera is presented in a way suggesting evaluation by others (as opposed to observation by an intimidating authority figure; see hypothesis 1). Thus, in addition to stimulating pro-social behavior, people intent on generating a positive image to the outside world would most likely show less undesired behavior as well. This leads to the following hypotheses:

*Compared to a control situation without camera presence, camera surveillance instilling the impression that others are watching/evaluating one's behavior will lower incidences of cheating (2a) and increases pro-social behavior (2b)*.

The third line of reasoning in which cameras could influence behavior focuses on interpersonal processes. This approach questions whether the probability of detection or the possibility of evaluation by others is actually an essential ingredient for behavioral change to occur. That is, research by Bateson et al. (2006) shows that a graphic representation of eyes suffices to prevent undesirable behavior. Specifically, when a picture of eyes was depicted in a university cafeteria (compared to a picture of flowers), a larger percentage of people paid for their drinks (i.e., donated money to an “honesty box”). What is most interesting about this study is that the image of eyes might have caused a sense of being watched, while no “real” others were involved, and thus no punishment or evaluation could be anticipated or foreseen.

Interestingly, Research by Govern and Marsch (2001) shows that not just the presence of a camera can make participants more self-aware, but that similar effects occur when participants are seated in front of a mirror (and hence, when there is no one watching but the participants themselves). Kallgren et al. (2000) reproduced this effect by letting participants watch themselves on a monitor, and observed that those participants did indeed litter less (so they adhered better to social and/or personal norms) compared to participants who did not watch themselves on a monitor.

In other words, focus on the self-image by means of mirrors or monitors can result in a stronger focus on norms and values present in the individual self (Duval and Wicklund, 1972; Carver and Scheier, 1978). Wicklund and Duval (1971) call this process self-evaluation, where people being observed reflect on their behavior and subject it to their personal norms and values, resulting in more normative behavior.

The present study will explore the possibility that watching oneself on a monitor makes participants more aware of their behavior and more focused on complying to existing or personal norms and values, even without the (suggested) presence of anyone who could evaluate behavior. This leads to the following hypotheses:

*Compared to a control situation without monitor, watching oneself on a monitor should decrease cheating behavior (3a) and enhance pro-social behavior (3b)*.

This condition is distinct from the two other camera conditions in respect that participant get direct feedback from the image, instead of being confronted with a stationary surveillance camera. This makes this condition comparable to studies using mirrors, as well as to the other camera conditions we use in this study, since we did use a camera in our monitor setup.

Many previous studies on the influence of camera surveillance or monitors/mirrors on behavior do assume -rather than test- mediating processes, such as self-representational motives or self-awareness. One reason for this is that these psychological processes may not necessarily take place on a conscious level and are therefore difficult to capture with an explicit questionnaire (e.g., priming; Cameron et al., 2012). Therefore, researchers have argued that psychological mechanisms might best be uncovered by examining the impact of meaningful moderating variables, by measuring or manipulating them (i.e., variables which may either enhance or inhibit observed patterns. An example of this methodology can be seen in the study by Greenberg et al., 1992). In the current study we will follow this line of reasoning by including three personality traits that may interact with the manner in which the presence of a camera or monitor is presented.

First, it has been suggested that individuals with an external (rather than internal) Locus of Control are more sensitive to external/environmental cues in general, and the influence of chance and powerful others in particular (Rotter, 1966). It thus seems feasible that the suggestion of authority influences people with an external LOC more strongly compared to people with an internal LOC.

Also, some people might be more sensitive to cues of being watched or being evaluated. Personality traits that might play a role with regard to this aspect are Need for Approval (NA) and Self-Monitoring (SM). People with high NA are more concerned about impression management, and will be more inclined to show “good” behavior and avoid “bad” behavior in front of others (cf. Van Rompay et al., 2009). Self-monitoring is conceptually close to NA. SM describes to which extent people are willing to adapt their behavior in order to get the approval of others (Gangestad and Snyder, 2000). So, participants who score high for NA or SM are expected to be more inclined to behave themselves properly in front of cameras.

Finally, and as discussed before, personal norms and values might be activated by means of self-evaluation (induced by seeing oneself), and some personality traits might play a role in the effect of cameras and self-focus on behavior. One of such traits we are interested in is social value orientation (SVO), because SVO indicates whether a person is more inclined to behave pro-socially or more egoistically (Messick and McClintock, 1968; De Dreu and Van Lange, 1995; Van Lange, 1999). If the presence of a camera or monitor activates personal norms, it is likely that the effect of SVO on actual behavior will be stronger in these conditions.

## Method

### Experimental design and conditions

As stated in the introduction, we have three lines of reasoning in which behavior could be influenced by the way a camera or a monitor is presented in the environment. We wanted to put these lines of reasoning to the test in a single experiment so we would be able to evaluate each method in the same context.

Our experiment consisted of three conditions with a camera present, which were each compared to a control condition without any camera or monitor present (“no camera present”). The first condition involved a surveillance camera, presented in a way that makes it clear that an authority is watching who is able to punish (“authority watching”). The second condition incorporated a surveillance camera which was presented in a way which suggests one's behavior is being evaluated by others (“evaluation by others”). In the third condition people were not being watched at all, but were merely watching themselves on a monitor (“self-observation”).

In the three conditions where a camera was present, each participant received a form they needed to sign which outlined the purpose of the camera in the room. This form informed the participant about the presence of the camera, the fact that the experimenter could not access any recorded material, and the advice that they should act natural despite the camera presence. With signing, they would give permission that other departments of the university could use the material. In reality, the only video material recorded was by a hidden camera, and all of it was only accessible by the experimenters.

One paragraph on the form differed per condition; this was an important part of our manipulation. In the condition “authority watching,” the following text was included: “These recordings will be viewed by an independent party to make sure there are no disturbances or punishable behavior occurring during the study.” For “evaluation by others”, the following text was included: “These recordings will be used in an unrelated study in which different types of nonverbal behavior will be encrypted. The behavior will be recorded throughout the study.” For the condition “self-observation”, the following text was included: “These recordings are used to register the eye movements of the participant. The recordings will be digitally encoded in real-time by a computer; the original video stream will not be saved.”

The participants were asked to solve nine puzzles. They were told that for every correctly solved puzzle they would earn money. Participants were told the reason for the experiment was to test whether people would solve more puzzles and be more positive about the task when a monetary reward was offered for solving each puzzle. During the experiment there was the possibility to cheat, which could be monitored by means of a hidden camera. At the start of the task they received a plastic cup with a preferred drink. During the task the experimenter “accidentally” dropped a can with pens. We measured pro-social behavior by observing three types of behavior: throwing away their empty plastic cup, helping the researcher collect pens that have fallen to the ground, and donating (some of) the money earned to a good cause after the experiment ended.

### Participants

A total of 86 Bachelor students of psychology and communication studies at the University of Twente in the Netherlands participated in our research for credits or a small fee. These participants varied in age from 18 to 30 years (mean age = 21.56, Standard Deviation [SD] = 2.29). The sample consisted of 30 men and 56 women. Participants were randomly assigned to the experimental conditions.

### Procedure

Respondents participated individually in the experiment; one participant at a time. The participant was welcomed in the room, which was decorated as an office, with two desks, a plant, and a bookcase with books and binders. Shortly after arriving, the participant was asked to sign an informed consent form, and they were offered something to drink. After the experimenter returned with the drink in a disposable cup, the participant received another form (which was an important part of the manipulation, disguised as an additional type of informed consent form), informing them about the presence and purpose of the camera or monitor in the room (note that in the no-camera condition, this briefing did not occur). They were asked to read this meticulously, and sign the form when they were finished reading. The experimenter was present during reading, and checked afterward whether the participant had understood the information correctly.

**Figure 1:**
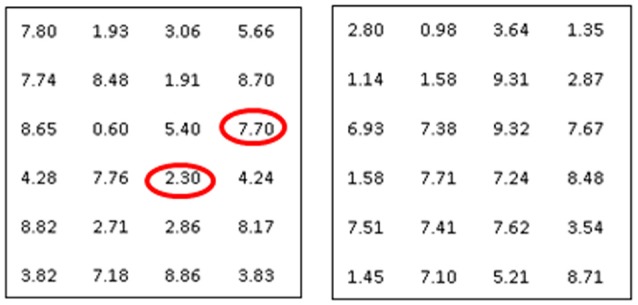
**Left**: solvable puzzle, **Right**: unsolvable puzzle.

After signing of the form, the experimenter gave the participant instructions for the main task, the puzzle task. For this task, participants had to figure out whether or not a matrix (containing 24 numbers in total) contained two numbers that added up to exactly ten. If they found these two numbers, the matrix had a solution which they could indicate by writing a big “+” with a marker on the answering form. If the matrix did not have a correct solution, they could write a “−“ on the answering form (see Figure 1). This test is based on a task designed by Mead et al. (2009), slightly adapted for this study (we included 24 instead of 12 numbers to increase the difficulty/time to solve the puzzles). Participants first read the instructions which were also explained by the experimenter, and then received a paper with nine matrices. As soon as they were handed the puzzles, a timer started counting down from 10 min, and the experimenter left the room. Before the experimenter left, she explained she had to do something and wouldn't be back before 15 min had passed, so she asked if the participant would stop working on the puzzles as soon as the alarm on the timer rang. The participant was instructed to get the form with the correct answers from the second desk in the room after the 10 min had passed, and check the correct answers against their own answers, so they could report back the number of correct answers to the experimenter as soon as she got back.

During this task, participants had the option to cheat in multiple ways: they could work longer than 10 min on the puzzles, they could correct their answers afterwards with the answers on the answering form, or they could copy additional answers from the answering form. Additionally, they could guess after time was up (filling in random solutions to the puzzles without checking).

After the experimenter returned, the participant was asked to give the complete solution form to the experimenter, and subsequently received another questionnaire (distraction and manipulation check). While the experimenter handed over this form, she “accidentally” knocked over a cup with pens, pencils and other office supplies on the desk, and observed whether or not the participant helped her collecting all the pens from the floor. After the participant got another moment alone to fill out these forms, the experimenter returned to debrief and pay the participant (in 50 cent coins), and give the participant an opportunity to donate a part of their earnings to the World Wildlife Fund (WWF). The experimenter had a donation box to this end, which she showed to the participant while making clear that any donation was completely voluntary and that it was a campaign of the WWF in which multiple researchers participated.

Twenty-four hours after the completion of the experiment, the participants received an email with a link to an online survey. They were told this questionnaire was unrelated to any previous participation in a study and it was included only to ensure the participants could actually earn a full credit for a whole hour of participation in experiments.

### Ethics statement

Prior to conducting this research this study was submitted to the Ethical Review Board of the University of Twente, department of Behavioral Sciences and has been approved. During the experiment, the participants were instructed and asked to sign a written consent form, which they all did. After signing they had the opportunity to ask questions. All participants were anonymized during the study and in the data files. After the experimental part the participant was briefly debriefed. After the study concluded all participants were send an email with a detailed explanation of the study.

### Manipulation checks

We included the following question in the last questionnaire during the experiment to check whether or not the participant understood the condition they were in: I was told that the camera is present in the room in order to: (a) prevent illegal and irregular behavior, (b) code my non-verbal behavior for an unrelated study, or (c) code my eye-movements digitally. The 2 participants who answered this question inconsistent with the assigned condition were excluded from further analyses.

### Measures

#### Behavioral outcome measures

The main dependent variables were different kinds of cheating and pro-social behavior, explained below.

#### Cheating

Participants could cheat in four different ways: they could copy answers from the answering form to the puzzles they didn't yet work on, they could correct their answers with the answers from the answering form, they could continue after time was up, or they could guess the answers to the puzzles they didn't yet work on after time was up. Although, nobody directly copied the answers from the answering form to puzzles they didn't work on, we observed several instances of the other types of cheating and guessing behaviors. Guessing happened most often, even though the instructions did clearly state the participants should try to solve each puzzle before writing down an answer and stop as soon as the time was up. Per participant, and for each of the three categories we first estimated whether it occurred or not (yes/no). Next, we established per participant whether they cheated (correct answers and/or continue after time was up) or guessed at the deadline.

#### Pro-social behavior

We measured three different kinds of pro-social behavior: whether they threw away their trash at the end of the experiment, whether or not participants helped the experimenter collect fallen pens, and whether they donated money (and how much) to a good cause. Throwing away trash and helping were both recorded as dichotomous variables. Donating money was recorded as a percentage of the participant's earning during the experiment, ranking from 0 to 100%.

### Personality traits as moderators

Twenty-four hours after participating in the experiment the participants received an e-mail with a link to a personality questionnaire (presented as part of another study). This questionnaire consisted of demographical questions and the following personality constructs: Locus of Control, Need for Approval, Self-monitoring and Social Value Orientation. Each one will be explained in detail below.

#### Locus of control

To measure locus of control we used a Dutch translation of the “Rotter Internal-External Control scale” (Rotter IE scale; Rotter, 1966). The Rotter IE scale consists of 23 items, each of which gives the participant the choice between two options; for example: (a) “When I make plans, I am almost certain that I can make them work” (internal LOC, score “0”) and (b) “It is not always wise to plan too far ahead because many things turn out to be a matter of good or bad fortune anyhow” (external LOC, score “1”). All answers are then counted, resulting in individual scores ranging on a scale between 0 (internal control) to 23 (external control).

#### Need for approval

To measure Need for Approval we used a Dutch translation of the Need for Approval scale developed by (Strahan and Gerbasi, 1972); see also Van Rompay et al. (2009). This scale consists of 20 items. An example of a question is: “I will always try to stay polite, even to people I don't like.” The items were measured on a scale ranging from 1 (“not at all agree”) to 6 (“very much agree”) (α = 0.66).

#### Self-monitoring

To measure Self-monitoring we used a Dutch translation of the Self-monitoring scale developed by Snyder and Gangestad (1986). This scale consists of 18 items. An example of a question is: “I can only defend the ideas I believe in.” The items were measured on a scale ranging from 1 (“not at all agree”) to 7 (“very much agree”) (α = 0.78).

#### Social value orientation

To measure Social Value Orientation we used a decomposed game measure (Messick and McClintock, 1968; Kuhlman and Marshello, 1975). This measure can be used to classify individuals in a group of prosocially vs. egoistically oriented people. It consists of a series of nine choices between distributions of outcome points, which were said to have value to both oneself as well as (hypothetical) another person. Participants were given a choice among three alternatives, each corresponding with a tendency to maximize joint outcomes (i.e., prosocials) or a tendency to maximize own outcomes either in an absolute sense (i.e., individualists) or relative to others' outcomes (i.e., competitors; De Dreu and McCusker, 1997; Van Lange et al., 1997).

An example of a task is: “I give: (a) myself 400 points, the other 400 points, (b) myself 500 points, the other 300 points, (c) myself 420 points, the other 380 points.” Based on this task, the participants were categorized in pro-social vs. pro-self (either individualistic or competitive), because research shows that people with individualistic and competitive orientations show very similar behavioral patterns (e.g., De Dreu and Van Lange, 1995; see also Giebels et al., 2003). Five participants out of 86 could not be assigned a dominant orientation based on six or more answers in one category.

### Analysis

To test the main effects of the camera-conditions and monitor-condition on cheating, we used Fisher's Exact Test, which is a variant of the Chi Square Test specifically suited for low N and frequencies of < 5 per cell (Field, 2009). The Fisher's Exact Test works well with small sample sizes, and it is known to be rather conservative (actual rejection rate of H_0_ is lower than α). We also used the Fisher's Exact Test and a Kruskal-Wallis test (a non-parametric ANOVA) to test the main effects of the camera-conditions and monitor-condition on pro-social behavior.

As an exploratory part of this study we wanted to test whether several personality traits interacted with camera condition. However, because of the small incidence of cheating in the present study, interactions are difficult to determine. Therefore, we used classification trees. Classification trees are especially suitable for exploratory data analysis. This method is mainly known for its use in data mining, but has shown its usefulness in many fields; including clinical data (Su et al., 2011), test performance data (Gao and Rogers, 2011) and ecological data (De'ath and Fabricius, 2000). While mostly being used with large datasets, this non-parametric method is also very well suited to analyze small datasets, especially where other methods fail because of low frequency categories (e.g., see Hayes et al., 2015). Decision trees divide samples into meaningful sub-groups using “yes” or “no” questions, and present this data in an easy-to-read visual format. Theoretically, classification could continue until the end-nodes consist of only one case, but usually the division of the nodes is stopped when the improvement of the model doesn't increase by a reasonable amount by asking further questions (Kingsford and Salzberg, 2008). Classification trees can be used to identify variables influencing the dependent variable and possible interactions between variables, and sort them by relevance. This information can subsequently be used in further research, in which such indications of possible interaction effects are used to formulate interaction hypotheses and to test for them specifically.

## Results

For the purpose of clarity, we first discuss the results and hypotheses concerning cheating behavior, followed by pro-social behavior.

### Cheating behavior: overview

In total, 10 of the 86 participants cheated, and 12 guessed answers after the time they were allowed to work on the puzzles was up. Table S1 in the Supplementary Materials illustrates how these numbers are distributed amongst the conditions, and gives an overview of all different types of cheating. The data we used in our analyses were “cheating overall,” and “guessing.” Table 1 below only shows the values used in our analyses.

**Table 1:** Number of participants who cheated/guessed in the different conditions.

	**No camera present (*N* = 21)**	**Authority watching (*N* = 20)**	**Evaluation by others (*N* = 22)**	**Self- observation (*N* = 23)**
Cheating overall	7	0	3	0
Guessing after time up	5	1	4	2

Although there seems to be a reasonable difference between conditions in number of participants who had guessed, a Fishers Exact Test does not yield any significant results upon testing (*n* = 86, *p* = 0.29). We will therefore focus our analyses on the cheating overall variable.

### Cheating behavior: testing our hypotheses

First we checked whether there was an overall significant effect of our manipulation on cheating behavior, using “cheating overall” as the dependent variable. We used a Fisher's Exact Test (the Freeman-Halton extension of the Fisher exact for a 2 × 4 contingency table), which yielded a significant difference of the manipulation (*n* = 86, *p* = 0.001). Pairwise testing shows that this could be explained by the difference between the conditions “authority watching” and “no camera present” and the difference between “authority watching” and “self-observation,” as explained in the next sections.

To test our hypothesis 1a we checked whether a camera presented in an authoritative manner would discourage cheating behavior. We compared the “authority watching” condition with the “no camera present” condition. The dependent variable we used was “cheating overall,” so both types of cheating behavior taken together. We used a Fisher's Exact Test, which yielded a significant difference between “authority watching” and “no camera present” (Fisher's Exact Test, *p* = 0.009). Participants cheated significantly less often in the “authority watching” condition compared to the “no camera present” condition, thus confirming our hypothesis 1a (see Table S1 for the exact numbers per condition). According to our results we can draw the conclusion that a camera presented in a way which suggests a watchful authority is keeping watch will discourage cheating behavior.

Hypothesis 2a states that a camera presented in a way suggesting the participant is being evaluated by a public/unknown person has a discouraging effect on cheating behavior. To test this we compared the “evaluation by others” condition with the “no camera present” condition. The dependent variable we used was “cheating overall.” A Fisher's Exact Test showed no significant differences between these conditions present” (Fisher's Exact Test, *p* = 0.162, odds ratio: 3.17). Since there was no significant difference in the amount of cheating between the “evaluation by others” condition compared to the condition without any cameras present, we could not confirm our hypothesis 2a. According to these results, a camera presented in a way which suggests to the subject he/she is being evaluated by others, does not discourage cheating. Interestingly, it should be noted that in this condition, all the observed cheating incidences concern the type where participants correct the answers from a sheet.

For hypothesis 3a we suggested a monitor would cause people to reflect upon their own behavior and thus behave better, so we expect to observe less cheating behavior when a monitor is present. To test this, we compared the “self-observation” condition with the “no camera present” condition. Again, the dependent variable we used was “cheating overall.” A Fisher's Exact Test showed a significant effect between “self-observation” and “no camera present” (Fisher's Exact Test, *p* = 0.003). Participants cheated significantly less often in the “self-observation” condition compared to the “no camera present” condition, which confirms our hypothesis 3a. Therefore, we can conclude that when participants can watch themselves on a monitor, they will display significantly less cheating behavior.

### Pro-social behavior: overview

Participants could engage in different kinds of pro-social behavior during the experiment: they could clean up their trash after the experiment ended, they could help the experimented to collect pens after she knocked over a container full of pens, and they could donate a part of their earnings during the experiment to a good cause. Table 2 illustrates how these numbers are distributed amongst the conditions. At first glance, and in accordance with our expectations, cleaning trash was lowest in the authority watching condition, while all participants helped with the fallen pens in the evaluation by others condition. Furthermore, the willingness to donate to a good cause was lowest in the no camera condition when there was no watching at all. However, these variations are not significant.

**Table 2:** Number of participants who did and did not help and threw away their trash in the different conditions, and the mean of the donation percentage per condition (percentage of earned money).

	**No camera present**	**Authority watching**	**Evaluation by others**	**Self-observation**
Cleaning trash (yes/no)	6/11	3/10	6/11	6/10
Help with pens (yes/no)	7/12	8/12	11/11	8/13
Donation (mean/SD)	44%/43	56%/44	51%/37	54%/42

*The differences in total N are due to some participants who did not have a drink (they could not leave a cup/tea bag as trash), or the dropping of the pens by the experimenter not working out as it should (e.g., the pens didn't drop at all)*.

### ***Pro-social behavior: testing*** our hypotheses

First we checked whether there was an overall significant effect of our manipulation on pro-social behavior. The dependent variables we used were “cleaning trash,” “help with pens” and “donation.” For “Cleaning Trash” we used a Fisher's Exact Test (Freeman-Halton extension), which did not yield a significant result. For “help with pens” we used a Fisher's Exact Test (Freeman-Halton extension), which did not yield a significant result either. For “donation” we first performed a normality test. A Shapiro-Wilk test showed the data had a non-normal distribution, so instead of a regular univariate analysis (ANOVA), we proceeded to use a Kruskal-Wallis test. This test showed no significant effect of condition overall (*p* = 0.801), Therefore, we did not proceed with pairwise comparisons.

Hypothesis 1b states we expect a camera presented in an intimidating manner will discourage pro-social behavior. To test this hypothesis we compared the “authority watching” condition with the “no camera present” condition. The dependent variables we used were “cleaning trash”, “help with pens” and “donation”; the three different kinds of pro-social behavior we measured. For “cleaning trash” we used a Fisher's Exact Test, which showed no significant differences between these conditions (Fisher's Exact Test, *p* = 0.691, odds ratio: 0.55). For “help with pens” we also used a Fisher's Exact Test, which again showed no significant differences between these conditions (Fisher's Exact Test, *p* = 1, odds ratio: 0.88). For “donation” we used a Kruskal-Wallis test, which also showed no significant differences between these conditions. Due to no significant differences in the amount of observed pro-social behavior between the condition “authority watching” condition compared to the “no camera present” condition, we could not confirm our hypothesis 1b. According to these results, a camera presented in a way to suggest an authority is keeping watch does not discourage (nor encourage) pro-social behavior.

For hypothesis 2b we expect the presence of a camera presented in a way suggesting the participant is being evaluated by a public / unknown person will encourage pro-social behavior. To test this we compared the “evaluation by others” condition with the “no camera present” condition. Again, the dependent variables we used were “cleaning trash” (Fisher's Exact Test, *p* = 1, odds ratio: 1), “help with pens” (Fisher's Exact Test, *p* = 0.531, odds ratio: 0.58) and “donation.” We did not find significant differences between the conditions for any of the dependent variables, therefore we could not confirm hypothesis 2b. There was no significant difference in the amount of pro-social behavior between the “evaluation by others” condition compared to the condition without any cameras present. Therefore, we can conclude that a camera presented in such a way to suggest the participant is being evaluated by others, will not encourage (nor discourage) pro-social behavior.

Hypothesis 3b suggests a monitor would cause people to reflect upon their own behavior and thus behave more according to (their) norms and values, so we expect to observe more pro-social behavior when a monitor is present. To test this we compared the “self-observation” condition with the “no camera present” condition. The dependent variables we used were “cleaning trash” (Fisher's Exact Test, *p* = 1, odds ratio: 0.9), “help with pens” (Fisher's Exact Test, *p* = 1, odds ratio: 0.95) and “donation.” We did not find significant differences between the conditions for any of the dependent variables. Participants did not show any more pro-social behavior in the “self-observation” condition compared to the “no camera present” condition, therefore we could not confirm hypothesis 3b. According to these results we can conclude that when participants can watch themselves on a monitor, they will not show more pro-social behavior.

### Classification trees: testing the moderators

A classification tree built with dependent variable “cheating overall” and as independent variables the personality traits LOC, NA, SM & SVO shows that for all conditions -except the “no camera” condition-, the condition the participant is placed in is the most important factor to predict if people cheat or not. “Authority watching” and “self-observation” are shown to be more effective than “neutral camera.” “Authority watching” and “self-observation” seem to be comparable in effectiveness. For the condition without camera, the tree shows that LOC score is an important measure to predict whether people cheat, with a cut-off point on (12.5), which lies in the “external LOC” area (defined as 11 and higher), indicating that people with an internal Locus of Control (LOC score ≤ 12.5) are more inclined to cheat when there is no camera around, compared to people with an external locus of control (see Figure S1).

The best fitting classification trees for the pro-social behavioral measures cleaning trash and helping did not show any interactions between personality measures and the experimental conditions.

## Discussion

The purpose of this study was to examine whether displays of undesired behavior (cheating) and pro-social behavior (cleaning up trash, helping behavior and donating money to a good cause) are affected by the way indoor surveillance-methods are presented (i.e., framed). In most experiments, a situation with a camera present is pitted against a “no camera present” condition, however, different variations in framing of camera presence have to our knowledge never been tested in a single setup. We explored three different perspectives: the first approach is derived from criminology where we present the camera in a manner suggesting an authority who can punish is watching, an approach assuming undesired behavior is a rational process of cost-benefit analysis. The other two approaches stem from psychology; one assumes people try to represent themselves better in the real or suggested presence of others, the other approach assumes people will act in accordance with internally present norms and values when they are confronted by an image of themselves. We wanted to test these different methods of presenting surveillance in a single controlled experimental setting, in order to demonstrate that the “framing” of camera surveillance indeed makes a difference.

Our results support the popular notion that camera presence decreases undesired behavior, in this case: cheating. Our study also supports the idea that the framing of a camera's presence does indeed influence cheating behavior in different ways. The presence of a camera presented in a non-intimidating, non-authoritative manner managed to reduce cheating behavior slightly, but not significantly. However, cheating behavior decreased significantly when the camera was presented in an intimidating, authoritative manner. This supports the statement by Levine (2000) that the impression about the person who is watching through the camera is important for effects of camera surveillance to crystalize.

We found that a camera presented in an authoritative manner and a monitor facilitating self-observation are both significantly effective in the prevention of cheating behavior. Apparently not only the chance of being caught by an authority can be a great motivator to discourage cheating, but observing one's own image “does the trick” just as well. This is notable, because very different psychological processes are assumed to be responsible for these two pathways. In other words, both rational considerations may be involved (i.e., when an authority is watching, who can punish in case of misbehavior; cf. Levine, 2000), but also more implicit self-confrontational processes, enhancing a sense of a “just” self.

This study focuses on an indoor setting, where participants complete a typical task for this type of setting. In outdoor environments, a wider variety of environmental factors could play a role. For instance, people can more easily overlook the camera and its framing in an outdoor environment, whereas camera presence and accompanying messages could not be overlooked in our research. Hence, it would be worthwhile to test whether framing effects (such as those revealed in the current research) uphold in outdoor settings.

Looking at the type of cheating, we see that the type of cheating which is most affected by camera presence is “continuing after time runs out.” We categorized this as being the “less serious” type of cheating (as is evident from the frequency of occurrence in the “no camera” condition), but on the other hand, this is the type of cheating that is most easily caught on camera (i.e., more so than subtly correcting an answer upon checking the answers from the correction sheet behind the barrier on the desk). As we can see in the frequency table (Table 1), “continuing after time is up” did not occur with any type of camera present, while correcting did happen a few times when the camera was being neutrally presented in a non-intimidating manner. This might indicate that (in a camera's presence) detectability of undesired behavior is a more important factor than the seriousness of behavior, a finding which also points at rational processes playing an important role.

We did not find a significant effect of the presence of a camera or monitor on the occurrence of guessing. We measured the amount of guessing because we instructed participants to solve the puzzles, and guessing the answer could be considered as (mildly) transgressive behavior. However, guessing was not explicitly forbidden, and since guessing is a technique often used by students to answer multiple-choice questions, it will probably not be seen as deviant or undesired behavior during an experiment. This might explain why camera presence and framing did not affect this type of behavior.

While looking at pro-social behavior we did not find any significant results even though we did expect that the authoritatively presented security camera would discourage pro-social behavior. This might be attributed to the experimental setting in an university office building. The study by Van Bommel et al. (2014) suggested that the adverse effect of camera surveillance on helping behavior only occurred when no bystanders were present. Therefore, this adverse effect is more feasible to occur in an empty street setting compared to an indoor office setting, since an office setting most likely has bystanders present nearby. While we did have no bystanders present during our experiment, the possible presence of people nearby might have negated the effect. Additionally, the helping behavior we used in our experiment was very different from the type of helping behavior used in the study by Van Bommel et al. (2014).

When a camera was presented in a neutral, friendly manner we expected to see more pro-social behavior, in line with Van Rompay et al. (2009) who showed that people are more willing to help in an indoor setting when a camera is present. However, in the latter study the “accident” where the participant could help out the experimenter occurred at the beginning of the experiment, whereas in our study it took place near the end of the session. Arguably, at the beginning of an experiment, participants might be trying to make a good impression by representing his/herself well, and additionally might have been more sensitive to the new environment and the presence of the camera in particular.

We also expected to see more pro-social behavior when a monitor was present, because we expected the monitor to make people more focused on themselves and evaluate their own behavior, and thus behave more “correctly.” However, the presence of a monitor only seems effective in preventing undesirable behavior, while it does not seem to stimulate pro-social behavior. Previous research (Gibbons and Wicklund, 1982) indicates that situational factors (like the salience of the helping norm and the focus of attention) are highly relevant in predicting whether or not helping behavior is stimulated or impeded by self-focus via various means (e.g., by a mirror or listening to one's own recorded voice).

Explorative analysis of our data using regression trees hinted at potentially interesting interactions between camera presence and framing on the one hand, and personality traits on the other. Admittedly, this part of our study is explorative, designed to reveal possible personality-environment interactions influencing cheating behavior in our data, which should be further examined in larger-scale follow-up studies. The aim of this analysis was to bring some insight in how the different variables we measured and manipulated relate to each other and to cheating. Personality traits did not seem to interact with the conditions where a camera or monitor was actually present; those effects were best explained by the main effects already described. However, when there was no camera present (the “no camera condition”), the personality measure “Locus of Control” did seem to play a role in whether or not participants would cheat during the experiment. Specifically, participants with a more internal LOC were more inclined to cheat when there was no camera present. As people with an internal LOC can generally be seen as people who believe that the outcome of events result primarily from their own doing, this may explain why they will also seize the opportunity to earn more money on an experiment by cheating when they believe nobody is watching them.

The personality traits Need for Approval (NA) and Self-Monitoring (SM) did not seem to moderate the effect of camera surveillance on behavior. These traits are similar; both relate to leaving a good impression on others. We did not find an effect of these traits on the behavior of the participants being watched with a camera or watching themselves via a monitor, therefore these traits do not seem to be relevant in changing behavior in the conditions of our experiment. The last personality trait we explored is social value orientation (SVO), which regards a person's affinity to behave more pro-social or more pro-self. We theorized this trait might have been amplified by triggering self-evaluation, but this does not seem to be the case.

Concluding, this study shows that the way camera surveillance is presented is a primary factor to reckon with when employing camera surveillance as a means to guide behavior and prevent transgressions. Taking this into account might enable policy makers to increase the effectiveness of indoor camera surveillance in a low-cost manner (e.g., via visual communications, explicit verbal statements; i.e., audio messages). Additionally, this finding also sheds light on the fact why previous meta-analyses of camera surveillance often show contradictory results; context is all-important.

Furthermore, this study revealed that making people more focused on themselves by means of a monitor works equally well compared to the most effective camera condition. This could be taken to suggest that even without cameras or monitors, people can still be discouraged to show undesired behavior by very subtle means that lead people to reflect on what is just and correct. In addition to confronting people with their own image, future studies could explore to what extent watchful eyes presented on, for instance, posters in lobbies, entrances or waiting areas of indoor settings could help create the right mindset so that transgressions on subsequent tasks (e.g., filling out forms at the town center) might be counteracted. Awaiting future research exploring these and related issues, we feel confident that current undertaking opens up avenues for further integrating the fields of criminology and the behavioral sciences so as to make the best of situational crime prevention in the years to come.

## Author contributions

AJ was involved in the design of the study, analysis and interpretation of data, and drafted the manuscript. EG and TvR were involved in the design of the study, reviewed all aspects of data collection, analysis and interpretation, and revised the manuscript critically for important intellectual content. MJ was involved in the interpretation of data and revised the manuscript critically for important intellectual content. All authors read and approved the final manuscript, and agreed to be accountable for all aspects of the work.

### Conflict of interest statement

The authors declare that the research was conducted in the absence of any commercial or financial relationships that could be construed as a potential conflict of interest.

## Supplementary material

Figure S1 A classification tree with the dependent variable “cheating overall,” and as independent variables the camera conditions (manipulation) “no camera,” “neutral camera,” “authority watching,” and “self-observation,” plus the personality traits LOC, locus of control; NA, need for approval; SM, self-monitoring; SVO, social; value orientation.

Table S1 In total, 10 of the 86 participants cheated, and 12 guessed answers after the time they were allowed to work on the puzzles was up. This table illustrates how these numbers are distributed amongst the conditions, and gives an overview of all different types of cheating. As can be derived from the table below, two participants used more than one type of cheating and four participants both cheated and guessed, of which one participant (in the condition without camera) used two methods of cheating and guessed during the experiment.
