# Supplementary figures and images for: The Influence of the Presentation of Camera Surveillance on Cheating and Pro-Social Behavior
Source: Front Psychol. 2018 Oct 16;9:1937. doi: 10.3389/fpsyg.2018.01937 (PMC6198084; doi:10.3389/fpsyg.2018.01937)

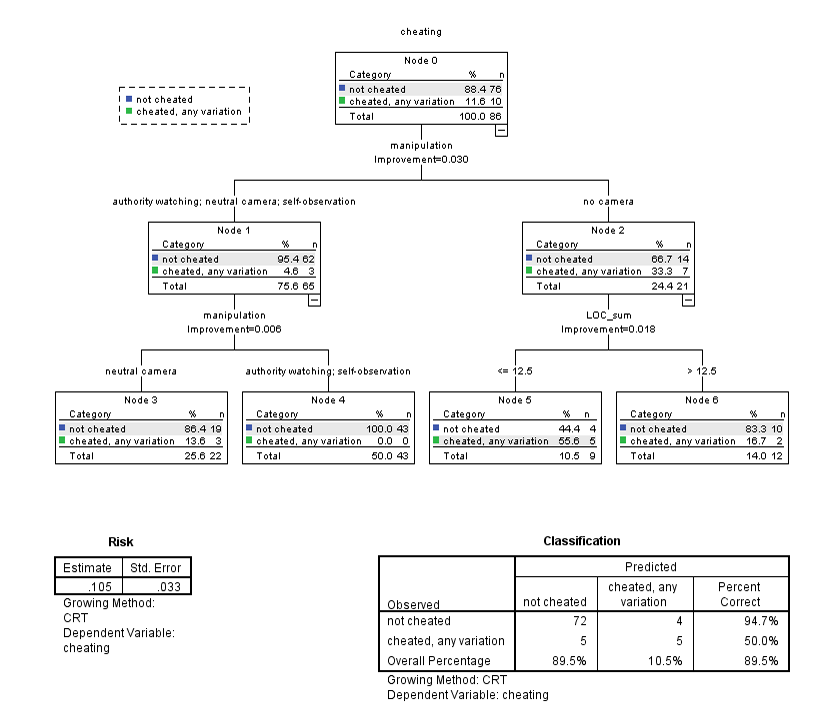

Supplement: Figure S1 — A classification tree with the dependent variable “cheating overall,” and as independent variables the camera conditions (manipulation) “no camera,” “neutral camera,” “authority watching,” and “self-observation,” plus the personality traits LOC, locus of control; NA, need for approval; SM, self-monitoring; SVO, social; value orientation. [file Image_1.TIF]
