# Supplementary material for: The Influence of the Presentation of Camera Surveillance on Cheating and Pro-Social Behavior
Source: Front Psychol. 2018 Oct 16;9:1937. doi: 10.3389/fpsyg.2018.01937 (PMC6198084; doi:10.3389/fpsyg.2018.01937)
Supplement: Table S1 — In total, 10 of the 86 participants cheated, and 12 guessed answers after the time they were allowed to work on the puzzles was up. This table illustrates how these numbers are distributed amongst the conditions, and gives an overview of all different types of cheating. As can be derived from the table below, two participants used more than one type of cheating and four participants both cheated and guessed, of which one participant (in the condition without camera) used two methods of cheating and guessed during the experiment. [file Table_1.docx]

Table S1: Number of participants who cheated/guessed in the different conditions.

|  | No camera present (N=21) | Authority watching (N=20) | Evaluation by others (N=22) | Self-observation (N=23) |
| --- | --- | --- | --- | --- |
| Cheating: continuing after time up | 6 | 0 | 0 | 0 |
| Cheating: correcting answers from sheet | 3 | 0 | 3 | 0 |
| Cheating during experiment (any kind) | **7** | **0** | **3** | **0** |
| Guessing after time up | 5 | 1 | 4 | 2 |
| Cheating AND/OR guessing during experiment | **9** | **1** | **6** | **2** |
| Note: The ‘overall’ data is not a sum of the frequency of the different types of cheating; a single participant could cheat in multiple different ways during the experiment.  ‘Cheating AND/OR guessing overall’ is not used in calculations; it is included in this table to illustrate how many participants used cheating and/or guessing during the experiment. | | | | |
